# Enhanced antibacterial effect of a novel *Friunavirus* phage vWU2001 in combination with colistin against carbapenem-resistant *Acinetobacter baumannii*

**DOI:** 10.1038/s41598-022-06582-0

**Published:** 2022-02-16

**Authors:** Phitchayapak Wintachai, Narumon Phaonakrop, Sittiruk Roytrakul, Ampapan Naknaen, Rattanaruji Pomwised, Supayang Piyawan Voravuthikunchai, Komwit Surachat, Duncan R. Smith

**Affiliations:** 1 School of Science, Walailak University, Thasala, Nakhon Si Thammarat 80161 Thailand; 2 Functional Proteomics Technology Laboratory, Functional Ingredients and Food Innovation Research Group, National Center for Genetic Engineering and Biotechnology, National Science and Technology Development Agency, Khlong Luang, Pathum Thani 12120 Thailand; 3 Division of Biological Science, Faculty of Science, Prince of Songkla University, Hat Yai, Songkhla 90110 Thailand; 4 Center of Antimicrobial Biomaterial Innovation-Southeast Asia and Natural Product Research Center of Excellence, Faculty of Science, Prince of Songkla University, Hat Yai, Songkhla 90110 Thailand; 5 Molecular Evolution and Computational Biology Research Unit, Faculty of Science, Prince of Songkla University, Hat Yai, Songkhla 90110 Thailand; 6 Institute of Molecular Biosciences, Mahidol University, Phuttamonthon, Nakhon Pathom 73170 Thailand

**Keywords:** Drug discovery, Microbiology

## Abstract

The emergence of carbapenem-resistant *Acinetobacter baumannii* (CRAB) has been increasingly reported, leading to greater challenges in treating infections. With the development of phage therapy and phage-antibiotic combinations, it is promising to improve the treatment of bacterial infections. In the present study, a novel vB_AbaP_WU2001 (vWU2001) phage-specific CRAB with a genome of 40,792 bp was isolated. Genomic analysis disclosed that it belongs to the *Autographiviridae* family of the order *Caudovirales*. Phage vWU2001 had a broad host range with a high adsorption rate, short latent period, large burst size and good stability. The phage could reduce preformed biofilms and inhibit biofilm formation. The combination of phage vWU2001 and colistin had significantly higher bacterial growth inhibition activity than that of phage, or colistin alone. The efficacy of the combined treatment was also evaluated in *Galleria mellonella*. Evaluation of its therapeutic potential showed that the combination of phage and colistin resulted in a significantly greater increase in *G. mellonella* survival and in bacterial clearance, as compared with that of phage or colistin alone, indicating that the combination was synergistic against CRAB*.* The results demonstrated that phage vWU2001 has the potential to be developed as an antibacterial agent.

## Introduction

*Acinetobacter baumannii*, an important pathogen in the ESKAPE group, is a gram-negative coccobacillus that is associated with both nosocomial and community-acquired infections. *A. baumannii* causes a variety of serious infections such as bacteremia, pneumonia, ventilator-associated pneumonia, meningitis, urinary tract infections, septicemia, wound infections, and central nervous system infections, resulting in high levels of morbidity and mortality. *A. baumannii* can form biofilms on biotic and abiotic surfaces, increasing bacterial survival and persistent infections^1,2^. Biofilms significantly decrease bacterial susceptibility to antibiotics, and they enhance the resistance and pathogenicity of *A. baumannii*. The development of *A. baumannii* resistance to a broad spectrum of antimicrobial agents, including carbapenems has been reported^3^. Carbapenem agents are often used to treat gram-negative infections, and carbapenem-resistant *A. baumannii* (CRAB) has been detected in intensive care unit patients worldwide^4–6^. CRAB is now becoming almost impossible to treat and the only available antibiotic capable of treating infections caused by CRAB is colistin^7^. However, colistin treatment produces renal and neurological side effects, and colistin at higher doses can increase the risk of nephrotoxicity and neurotoxicity^8,9^. Overuse and misuse of colistin in both humans and animals have led to emerging bacterial resistance^10^. Reducing antibiotic dosing and finding alternatives to antibiotics should be considered.

Bacteriophages (phages), viruses that infect and replicate in bacterial cells, are considered to be an alternative treatment in the post-antibiotic era^11,12^. Moreover, phages might be used in combination with antibiotics to improve their efficacy^13^. Phages are the most abundant organisms in nature and the high specificity of phages to host bacteria is an advantage. Many studies have been performed to develop phages as therapeutic agents for controlling bacterial infection, but only a few commercial phage products have been provided such as the Russian phage cocktail^14^, a pyophage cocktail from Georgia^15^, and phage products against food pathogens in USA^16^. Phages against *A. baumannii* have been characterized in vitro and in vivo such as phage ISTD against CRAB infection in vitro^17^, phage SH-Ab15519 against CRAB infection in vitro and in mice^18^, and phage Βϕ-R2096 against CRAB in *G. mellonella* larvae and a mouse model of acute pneumonia^19^. A combination of phages and antibiotics may increase therapeutic efficacy, such as phage vB_AbaM-KARL-1 and antibiotics which have a synergistic effect^20,21^. The combination of antibiotics and a 5-phage cocktail resulted in a significant reduction in *A. baumannii* biofilms^22^. The successful treatment of a multi-drug resistant (MDR) *A. baumannii* infected patient with a phage cocktail has been reported, with the MDR *A. baumannii* infection eliminated and the patient recovering^23^. This case caused an increase of interest in the development of phage therapy for patients. Recently, the combination of phage, tigecycline, and polymyxin E resulted in the clearance of CRAB infection in a pneumonia patient^24^.

Thus, the aim of this study was to isolate a new phage that was specific for CRABs*.* Physical and biological characterization of the phage such as determination of the host range, efficiency of plating, phage morphology, phage adsorption, one-step growth curve, lytic activity, phage stability under various temperatures, pH values, UV radiation and long term storage, genome analysis, and antibiofilm activity were determined. The combination efficacy of phage and colistin in contrast to a control CRAB infection was evaluated in vitro and *G. mellonella* larvae were used as an in vivo model*.*

## Results

### Phage isolation, purification, and virion morphology

**Figure 1:**
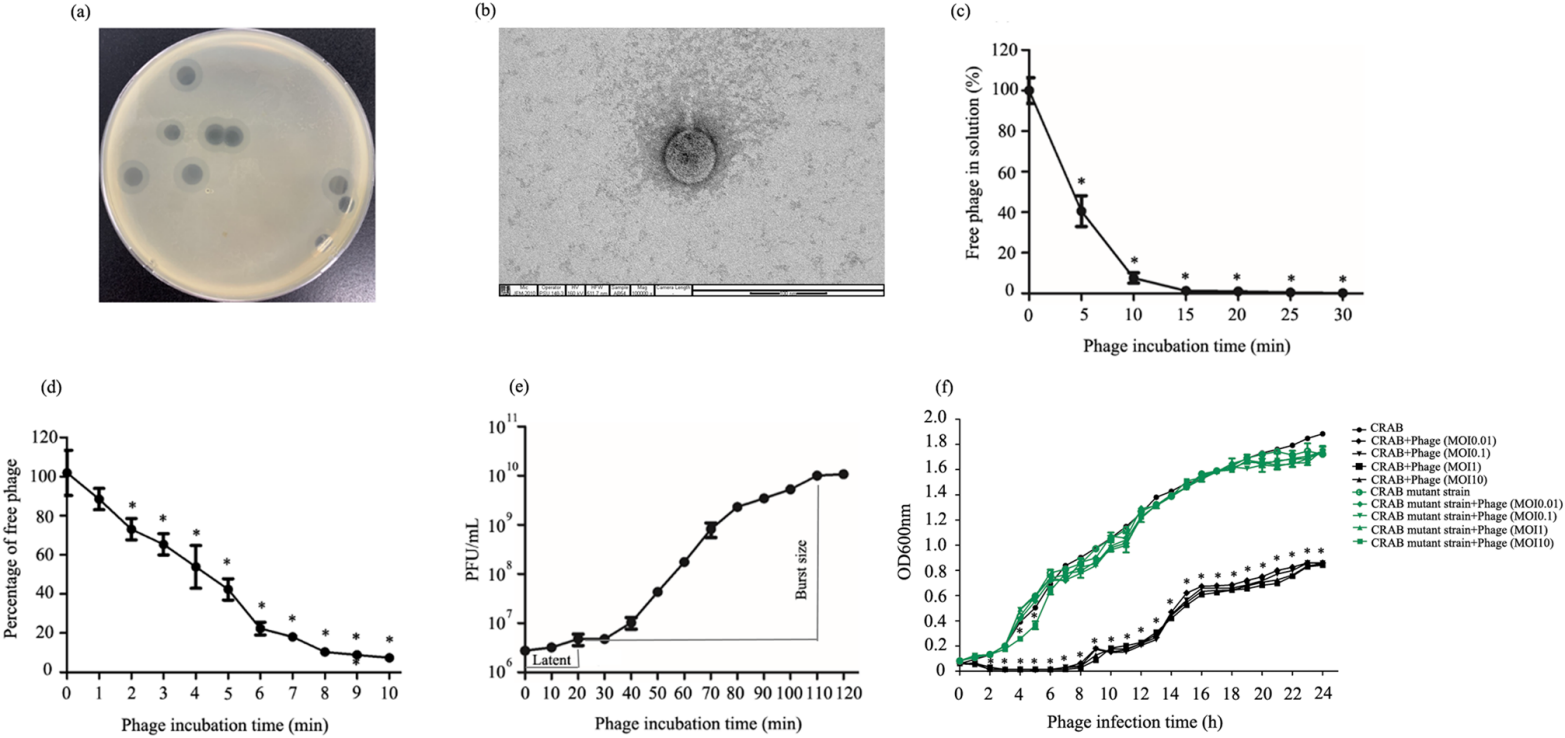
Phage isolation and biological characterization of phage. (**a**) Plaque morphology of phage vWU2001; (**b**) TEM of phage vWU2001; (**c**) adsorption velocity of phage vWU2001; (**d**) adsorption kinetics of phage vWU2001 to cells; (**e**) one-step growth curve of phage vWU2001; (**f**) lytic activity of phage vWU2001. Experiments were undertaken independently in duplicate with duplicate assay. The data show the mean ± SD (*, *P* value < 0.05).

A phage was enriched and then isolated from hospital wastewater samples after the wastewater treatment process. The phage produced large clear plaques 3–5 mm in diameter surrounded by a translucent halo on a CRAB lawn (Fig. 1a). The phage possessed an icosahedral capsid with a diameter of 70.62 (± 8.41) nm from vertex to vertex, and a short stubby tail (n = 3) (Fig. 1b). The morphological characteristics indicated that the phage vWU200 had a podovirus morphology. The phage was named phage vB_AbaP_WU2001 or phage vWU2001 based on the binomial nomenclature of bacteria viruses^25^. Following the Kropinski system, the prefix vB indicates a bacterial virus, Aba denotes *A. baumannii* and P denotes the morphological characteristic of *Podoviridae*. WU is an abbreviation of the university name, and the number “2001” denotes the year that the project was granted (2020) and the number of the phage (01), respectively.

### Phage host range and EOP

*Acinetobacter* phage vWU2001 susceptibility was determined by the spot test against 42 clinically isolated CRABs*, A*. *baumannii* ATCC 17978, *Escherichia coli, Klebsiella pneumoniae*, methicillin-resistant *Staphylococcus aureus* (MRSA)*,* and *Pseudomonas aeruginosa* strains*.* The spot test of phage vWU2001 showed that the phage had the capacity to lyse 54.76% of CRAB isolates (n = 42), indicating that phage vWU2001 might be a broad host range virus. There was no lytic activity of phage vWU2001 against *A*. *baumannii* ATCC 17978, a carbapenem-sensitive *A*. *baumannii* strain. Phage vWU2001 could not infect *E. coli, K. pneumoniae*, *MRSA,* or *P. aeruginosa.* To further assess the ability of phage vWU2001 to lyse CRAB, an efficiency of plating (EOP) assay was performed on 23 CRAB isolates that were susceptible to phage vWU2001. EOP is classified as high (EOP ≥ 0.5), moderate (EOP > 0.1– < 0.5), low (EOP ≤ 0.1) and no activity (EOP < 0.001) based on reproducible infection of the targeted bacteria. High (EOP = 0.56–1), moderate (EOP = 0.11–0.48) and low (EOP = 0.01–0.09) productive infection of phage vWU2001 were found for 11, 7, and 5 isolates, respectively (Supplementary Table S1).

### Phage adsorption

Adsorption kinetics of phage vWU2001 were measured. Phage adsorption onto CRAB cells occurred rapidly when compared to other phages such as *Acinetobacter* phage Aristophanes, a member of family *Autographiviridae*^26^ and *Escherichia* phage myPSH2311, a member of the *Podoviridae*^27^. Approximately 60% of the phage was adsorbed within 5 min. The adsorption reached more than 90% by 10 min post incubation. When the incubation time was increased, the free phage in solution continuously decreased (Fig. 1c). The adsorption rate constant *k* of phage vWU2001 was 7.23 × 10^–9^ ml/min (Fig. 1d).

### One-step growth curve

A one-step growth curve of phage vWU2001 was determined by infecting CRAB with phage. The latent period, the period between adsorption of phage to the host bacterial cell and the beginning of the release of new phage particles, was approximately 20 min (Fig. 1e). The calculated burst size of the phage, which is the ratio of the final phage concentration to the number of originally infected bacteria, was approximately 215 ± 46 PFU per bacterial cell.

### Lytic activity of phage against CRAB and the phage-resistant strain

To evaluate the efficacy of phage vWU2001 in controlling the growth of CRAB and a phage-resistant strain, lytic activity was determined through a killing curve. CRAB and the phage-resistant strain were infected with phage at MOIs of 0.01, 0.1, 1 and 10 followed by OD600 measurement. The results showed that the optical density (OD600) values of uninfected CRAB cells, as the control, increased continuously during incubation. In contrast, the absorbance of CRAB infected with phage vWU2001 at MOIs of 0.01, 0.1, 1 and 10 started to decrease gradually at 1 h post-infection (h.p.i.). At 2 h.p.i., the absorbance of phage infected CRAB was initially significantly lower than that of the control (*P* < 0.0001). After incubation for 6 h, the absorbance of phage infected CRAB increased (Fig. 1f).

The lytic activity of the phage against a phage-resistant strain was investigated. CRAB was infected with phage vWU2001 followed by incubation for 24 h. The supernatant was diluted and spread on TSA plates. Phage-resistant colonies were isolated, which exhibited a mucoid colony phenotype. A spot test was performed to confirm the phage resistant phenotype of the purified colony, and the bacteria was insensitive to phage vWU2001 infection, indicating that the selected colony was a phage-resistant strain. The efficacy of phage vWU2001 to control the phage-resistant strain was determined by a killing curve. The cell density of phage-resistant strain infected with phage vWU2001 at MOIs of 0.01, 0.1, 1 and 10 increased in parallel with uninfected phage-resistant strain. However, the growth of phage-resistant strain infected with phage vWU2001 at MOI of 10 was significantly slower than phage-resistant strain infected with phage vWU2001 at MOIs of 0.01, 0.1 and 1 at 4 to 5 h post infection (Fig. 1f).

### Stability of phage under various temperatures, pH values, UV radiation and long-term storage

**Figure 2:**
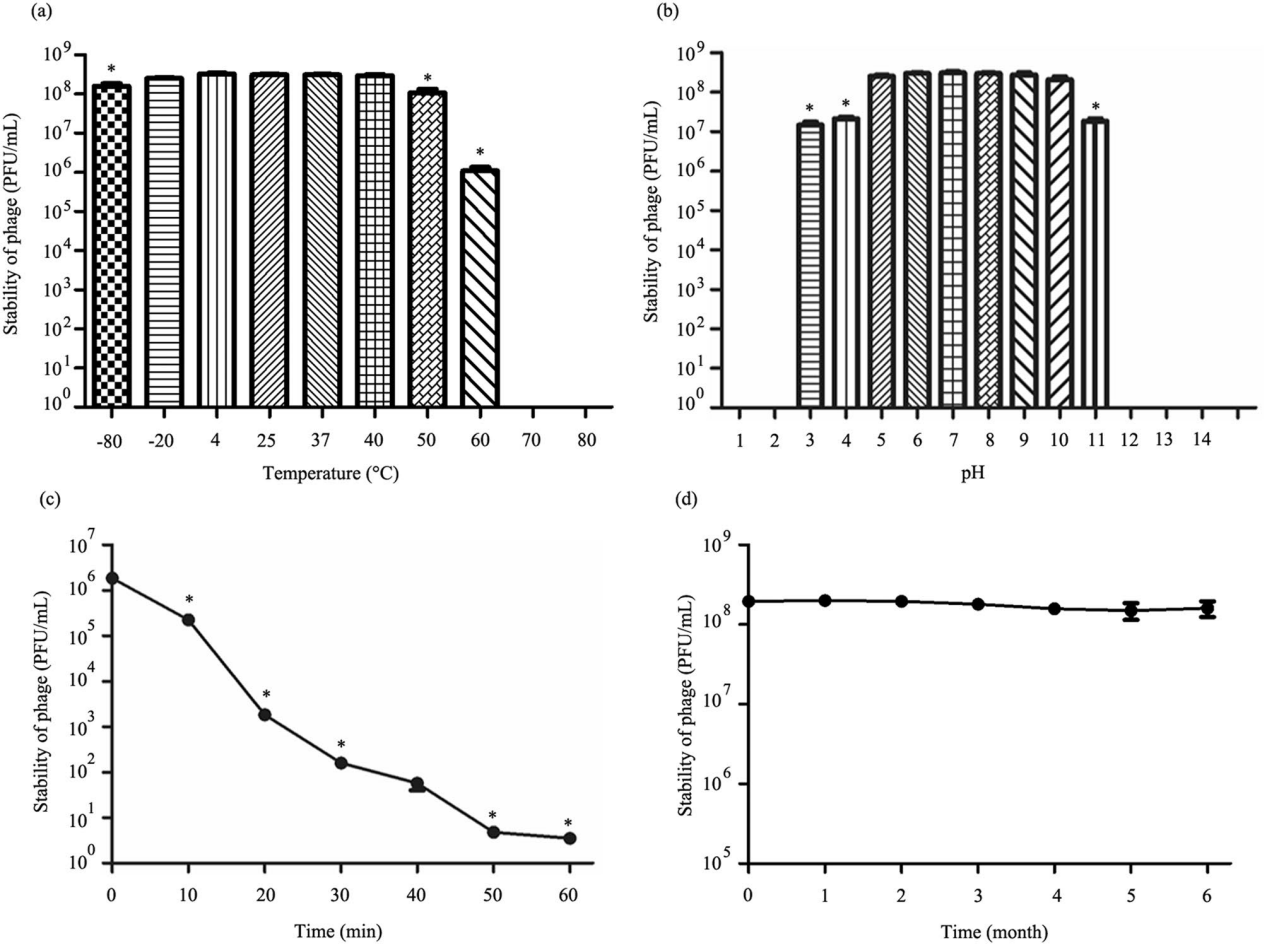
Stability of phage vWU2001. (**a**) Effect of temperatures on phage vWU2001 stability after 2 h of incubation; (**b**) effect of pH values on phage vWU2001 stability after 2 h of incubation; (**c**) effect of UV radiation on phage vWU2001 stability; (**d**) stability of phage vWU2001 under long-term storage at 4 °C. Experiments were undertaken independently in duplicate with duplicate assay. The data show the mean ± SD (*, *P* value < 0.05).

The stability of phage vWU2001 under different temperatures and pH values was evaluated. Thermostability tests were performed at different temperatures from − 80 to 80 °C. No significant change in phage stability was observed between − 20 and 40 °C. A significant reduction in phage lytic activity was observed at − 80, 50, and 60 °C (*P* ≤ 0.0182). No viable phage were observed at 70, and 80 °C (Fig. 2a). The stability of phage vWU2001 at − 20 and − 80 °C was also evaluated with different concentrations of glycerol, a cryoprotectant. There was no reduction of phage stability with 25% (v/v) and 50% (v/v) glycerol after storage at − 20 and − 80 °C for 7 days when compared with the stability of the phage at 4 °C, as a control (Supplementary Figure S1). The phage stability was monitored at pH 1 to 14. Following incubation, the phage had stable lytic activity between pH 5 and pH 10. A significant reduction in the lytic activity of the phage was observed at pH 3, 4, and 11 (*P* ≤ 0.0092) The phage lytic activity was completely lost at pH 1, 2, 12, 13, and 14 (Fig. 2b). The stability of phage vWU2001 under UV radiation was assessed. The phage viability was significantly reduced at 10 min post exposure (*P* ≤ 0.0042). At 60 min post exposure, the phage viability was reduced to less than 1% (Fig. 2c). For long-term storage, the stability of phage vWU2001 was monitored monthly for 6 months. There were no significant changes in phage viability (Fig. 2d). The results indicate that phage vWU2001 has good stability.

### Whole genome sequencing and bioinformatics analysis

**Figure 3:**
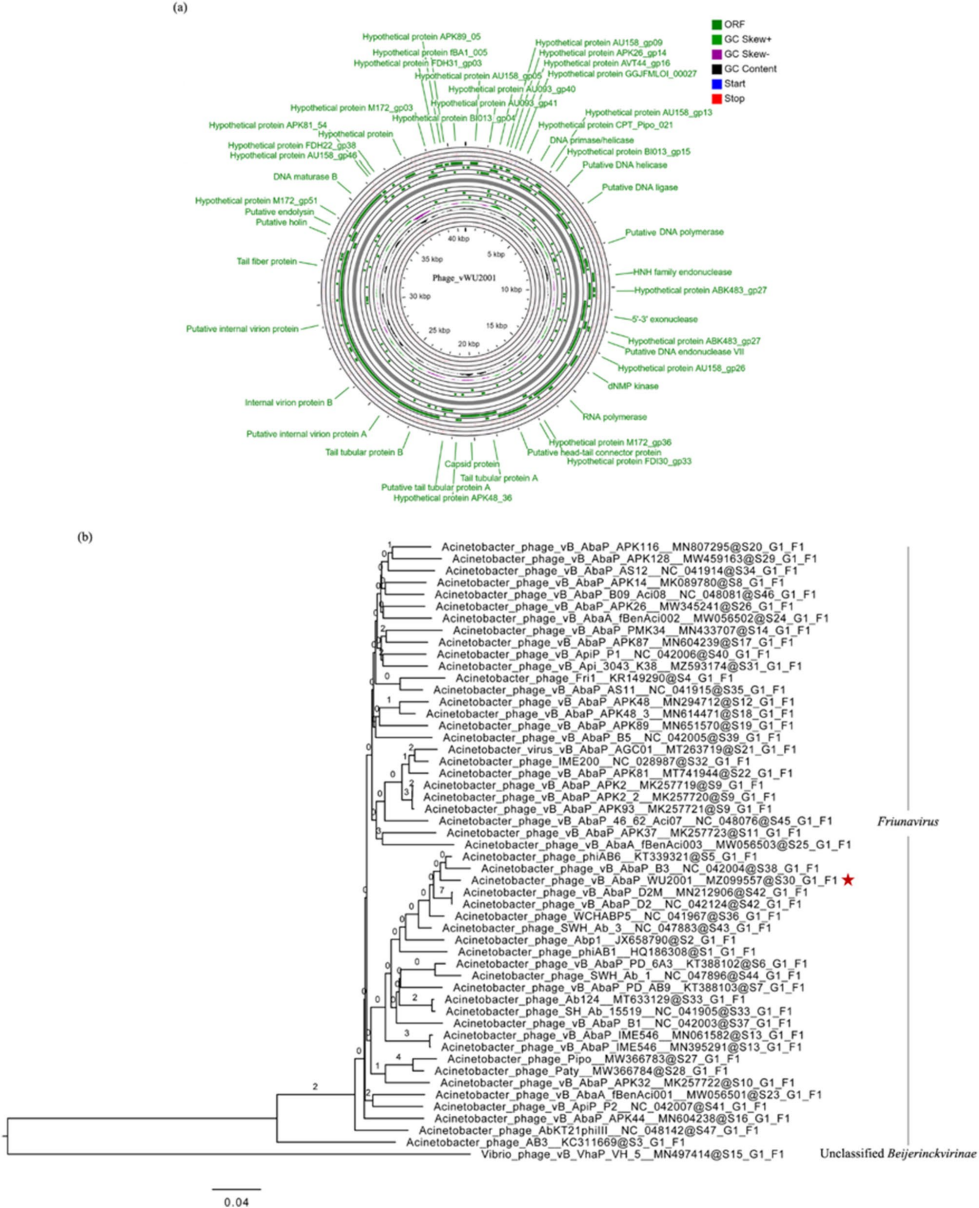
Phage vWU2001 whole genome analysis. **(a)** Map of the genome organization of phage vWU2001 created by the CGView program; (**b**) whole genome comparison of phage vWU2001 and other phages generated by the Genome-BLAST Distance Phylogeny (GBDP) method, with *Vibrio* phage vB_VhaP_VH-5 as the outgroup. Phage vWU2001 is highlighted with a red star.

The genome of the phage was sequenced using the Illumina sequencing platform followed by de novo assembly and annotation. The genome sequences of phage vWU2001 was deposited in the GenBank database with the accession number MZ099557.1. The raw reads have been deposited with the accession number SRR17460132. The phage genome was 40,792 bp in length with a G + C content of 39.4% (Fig. 3a). Genomic analysis revealed that the genome comprised of 47 predicted genes. The longest and smallest predicted genes encoded hypothetical proteins with 1032 and 37 amino acids, respectively. The sequences underwent a BlastX search to identify their functions. While 21 of the predicted gene products showed similarity to known proteins in phage, 25 were annotated as hypothetical proteins (Supplementary Table S2). One predicted gene had no significant similarity to the database so it was reported as a hypothetical gene of phage vWU2001. The genome of phage vWU2001 did not contain rRNA, tRNA, or tmRNA genes. Virulence genes and lysogeny-related genes were not found in the genome. Twenty-one gene products with predicted functions were classified into three main clusters: DNA replication/modification (DNA primase/helicase, putative DNA helicase, putative DNA ligase, putative DNA polymerase, HNH family endonuclease, 5’-3’ exonuclease, putative DNA endonuclease VII, dNMP kinase, RNA polymerase, and DNA maturase B), structural proteins (putative head–tail connector protein, tail tubular protein A, capsid protein, putative tail tubular protein A, tail tubular protein B, putative internal virion protein A, internal virion protein B, putative internal virion protein, and tail fiber protein), and host lysis (putative holin and putative endolysin). The proteins were also annotated using the Phyre2 web server (Supplementary Table S3). The results of twenty-one predicted proteins corresponded with the results of BlastX. Twenty-six predicted genes that were annotated as hypothetical proteins were categorized into 4 main functions; DNA replication (15 proteins), host infection (7 proteins), additional functions (3 proteins), and a de novo protein (1 protein). The genome of phage vWU2101 was also annotated using the RAST server (Supplementary Table S4). A large subunit of terminase (gp19) and a small subunit of terminase (gp18) that are responsible for DNA packaging were found in the genome^28^. Based on in silico prediction using PhageTerm analysis, terminal repeats on phage vWU2001 genome were predicted, However, PhageTerm provided ambiguous results for the first 1 to 25,000 nucleotides, so the prediction was based on nucleotides 25,001 to 40,792. To determine whether the level of sequence coverage might have impacted the PhageTerm analysis, the assembly coverage was analyzed by Geneious R10. The coverage of the whole assembly was × 4092, while the coverage of position 1 to 25,000 and position 25,001 to 40,792 was × 3,646.6, and × 4748.6, respectively, which is close to previously reported phage assembly coverage^29^, suggesting that the coverage is acceptable. However, it cannot be excluded that the lower coverage of positions 1 to 25,000 as compared to positions 25,001 to 40,792 may have affected the PhageTerm analysis of the whole assembly. The end of phage vWU2001 genome was redundant. The genome of phage vWU2001 had short direct terminal repeats (DTRs) of an estimated 397 bases from position 36,729 to 37,125, indicating that phage vWU2001 was classified as a DTR (short) phage based on the presence of few hundred basepairs of direct terminal repeats. Phage vWU2001 was classified as a DTR (short) phage in the same group as *E. coli* phage T7 (Supplementary Figure S2).

A phylogenetic tree analysis by ViPtree showed that phage vWU2001 was closely related to *Acinetobacter* phage phiAB6 (Supplementary Figure S3) and that phage vWU2001 was in a clade of *Acinetobacter* phage. The genome of phage vWU2001 was searched to identify the closest relatives using BlastN. The evolutionary relationships of phage vWU2001 with the relatives were analyzed the VICTOR virus classification and tree building online resources. *Vibrio* phage vB_VhaP_VH-5 (MN497414.1) was used as the outgroup. Phage vWU2001 was most closely related to *Acinetobacter* phage phiAB6 (KT339321.1, 94.87% identity) (Fig. 3b). Phage vWU2001 was also similar to *Acinetobacter* phage vB_AbaP_B3 (NC_042004.1), *Acinetobacter* phage vB_AbaP_D2M (MN212906.1), *Acinetobacter* phage vB_AbaP_D2 (NC_042124.1), *Acinetobacter* phage WCHABP5 (NC_041967.1) and *Acinetobacter* phage SWH-Ab-3. The results suggested that phage vWU2001 shoud be classified as a member of the genus *Friunavirus*, subfamily *Beijerinckvirinae*, family *Autographiviridae*, and order *Caudovirales*. The new species of phage was analyzed according to the criteria recommended by the International Committee on Taxonomy of Viruses (ICTV)^30,31^. The sequence of phage vWU2001 was subjected to BlastN search in NCBI to check the genome sequence identity, and was further analyzed for evolutionary relationship by determining the phylogenetic tree. In this analysis phage vWU2001 was closely related to *Acinetobacter* phage phiAB6. Genome-wide alignment of phage vWU2001 and *Acinetobacter* phage phiAB6, the closest relative phage were done by tBLASTx analysis, as inferred with ViPtree to assess genomic synteny. The results showed the conserved genomic syntenies and high identity within the genome (Supplementary Figure S4). However, less than 50% identity was detected in some parts of the genome. Whole genome percent identity was further analyzed by EMBOSS Stretcher that performed pairwise alignment of the two genome sequences. Phage vWU2001 shared 77.9% identity with *Acinetobacter* phage phiAB6. The previous report showed that phages were classified into the same species when the sequences identity was higher than 95%. Thus, the results indicated that phage vWU2001 was a new species in the genus *Friunavirus*.

**Figure 4:**
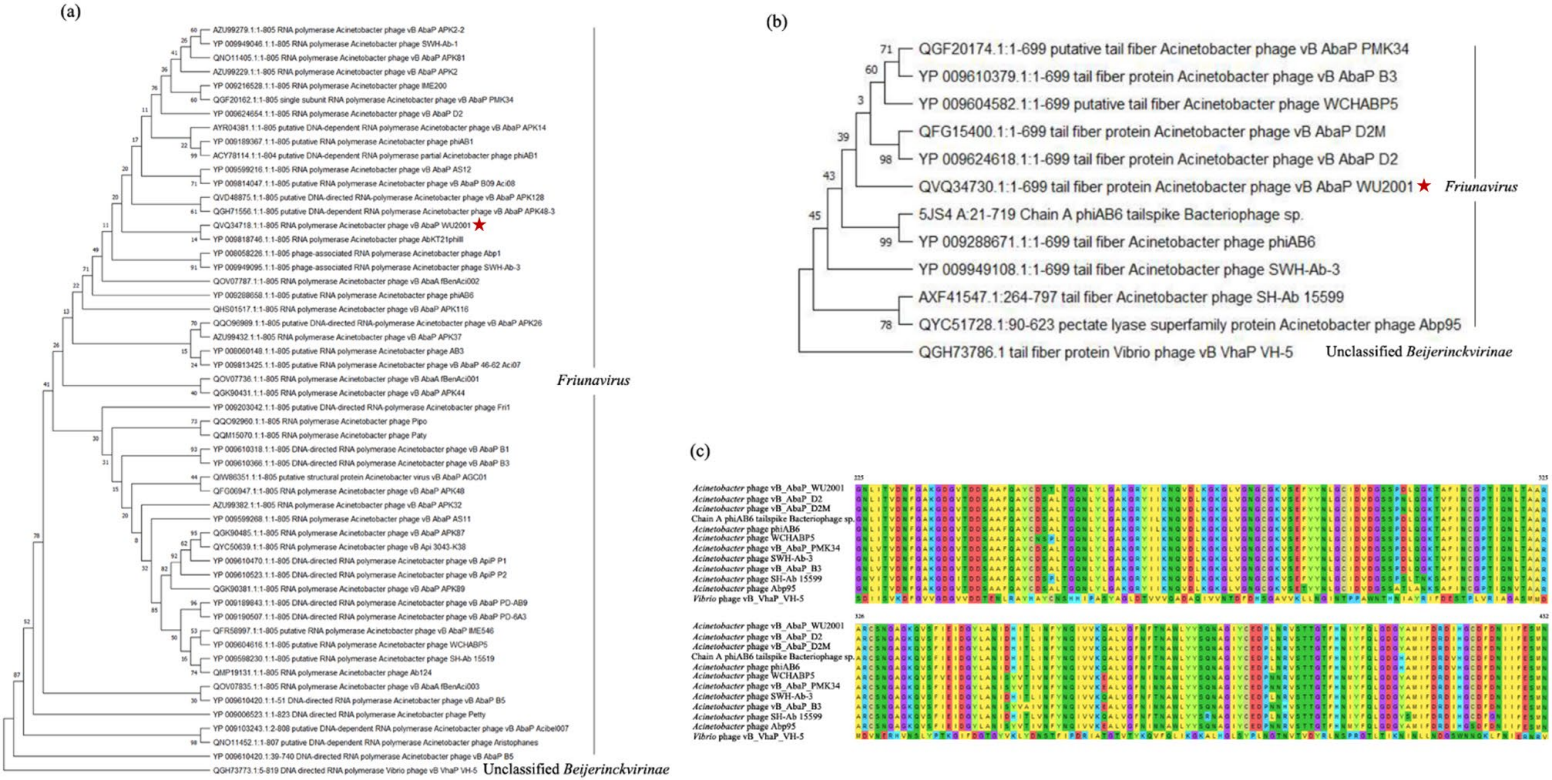
Phylogenetic tree and multiple sequence alignment analysis. Maximum-likelihood phylogenetic tree (JTT matrix-based model) based on the amino acid sequence alignment of the (**a**) RNA polymerase and (**b**) tail fiber. The multiple sequence alignments were performed with MUSCLE and the maximum likelihood phylogenetic trees were constructed in MEGA-X using 1000 bootstrap replicates. Numbers on nodes indicate the bootstrap support of the node. The RNA polymerase and tail fiber of *Vibrio* phage vB_VhaP_VH-5 were used as the outgroups, respectively. Phage vWU2001 is highlighted with a red star; (**c**) amino acid sequence alignment of the phage tail fibers. The sequences were aligned by MUSCLE in MEGA-X. Amino acid position 225–432 were selected to present.

Two specific genes, the RNA polymerase and the tail fiber, were selected to analyze the phage relationships. To select the phages for phylogenetic tree analysis, the nucleotide sequences of RNA polymerase and tail fiber were searched using BlastX against standard databases with the E-value cutoff of 0, the query coverage cutoff of 70%, and the identity cutoff of 60%. The related sequences were retrieved from the databases to construct the phylogenetic tree. The RNA polymerase of phage vWU2001 shared a common clade ancestor with the RNA polymerase of *Acinetobacter* phage AbKT21phiIII (YP_009818746.1, 99.13% identity) (Fig. 4a). Tail fiber binds specifically to the surface of the host bacterial strain, and differences in the tail fiber protein might affect host binding, so the phylogenetic tree of the tail fiber might provide insights to understanding the host range. The tail fiber of phage vWU2001 was grouped in the same clade and shared nucleotide sequence identity with the tail fiber of *Acinetobacter* phage vB_AbaP_D2 (YP_009624618.1, 99% identity), tail fiber of phage vB_AbaP_D2M (QFG15400.1, 98.71% identity), putative tail fiber of phage WCHABP5 (YP_009604582.1, 97.71% identity), tail fiber protein of phage vB_AbaP_B3 (YP_009610379.1, 96.57% identity), and putative tail fiber of phage vB_AbaP_PMK34 (QGF20174.1, 96.57% identity), respectively (Fig. 4b). Amino acid sequence alignment of the phage tail fibers showed the highly conserved sequences, but the differences between sequences were observed (Fig. 4c).

### Anti-biofilm properties

**Figure 5:**
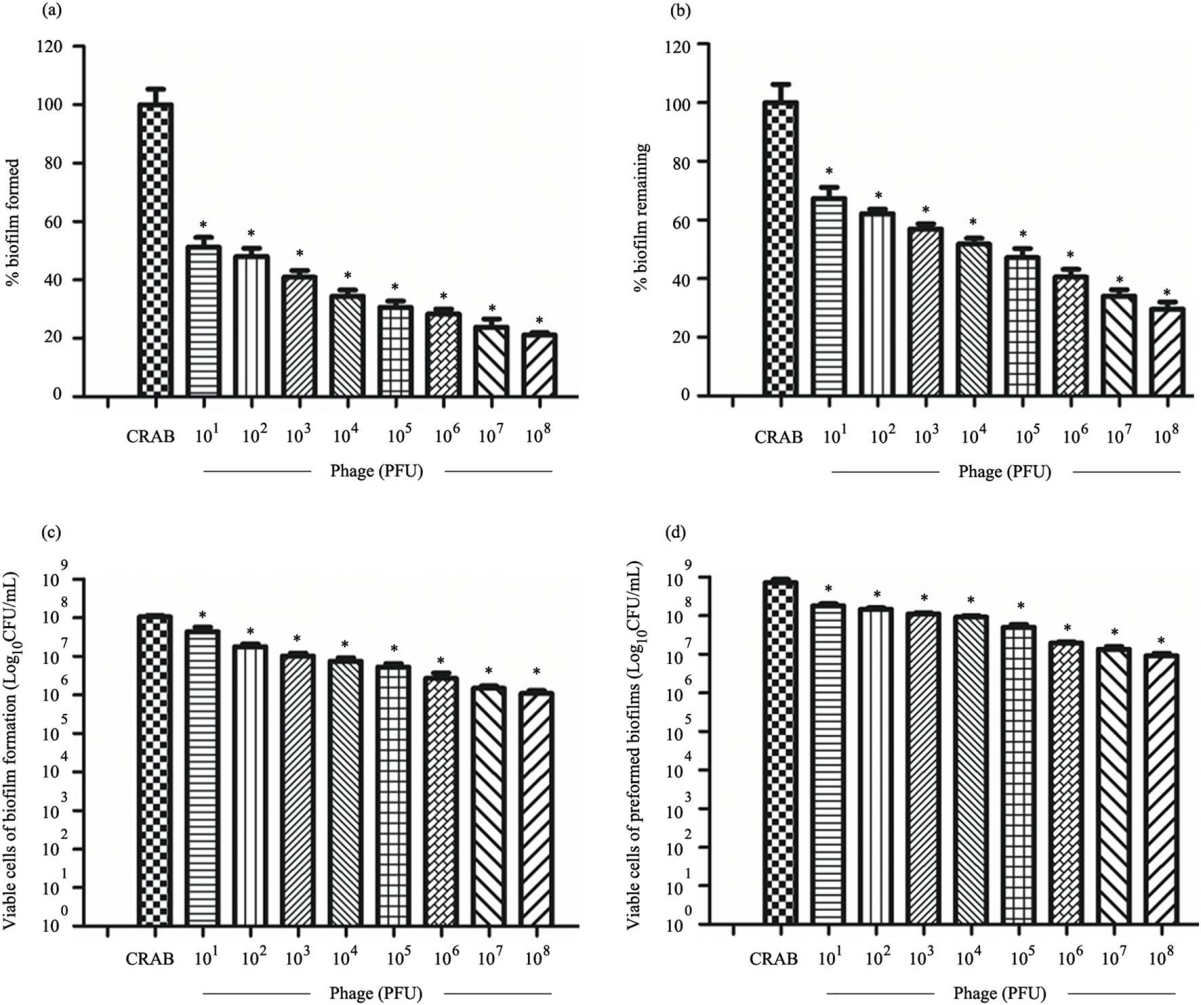
Effect of phage vWU2001 on CRAB biofilm formed in polystyrene surfaces. (**a**) Effect of phage on biomass of biofilm formation; (**b**) effect of phage on biomass of preformed biofilm; (**c**) effect of phage on viable cell numbers in biofilm formation; (**d**) Effect of phage on viable cell numbers in preformed biofilm. Experiments were undertaken independently in triplicate with duplicate assay. The data show the mean ± SD (*, *P* value < 0.05).

The efficacy of phage vWU2001 in reducing and preventing biofilms was investigated by crystal violet staining and cell viability counting. To reduce biofilm formation, CRAB was co-incubated with phage at various concentrations*.* Approximately 48.72 to 78.82% biofilm biomass, and 0.7 to 2 log of viable cells were reduced by phage vWU2001 at 10^1^ to 10^8^ PFU/well, respectively (*P* ≤ 0.0002 and *P* ≤ 0.0027) (Fig. 5a,c). For preformed biofilm removal activity of phage vWU2001, biofilms of CRAB were formed followed by phage treatment. Phage of 10^1^ to 10^8^ PFU/well significantly removed the preformed biofilm by 32.62 to 70.78% (*P* ≤ 0.0014) (Fig. 5b). At 10^1^ to 10^8^ PFU/well, the phage significantly decreased 0.5 to 1.8 log of viable cells (*P* ≤ 0.0038) (Fig. 5d). The results indicate that phage vWU2001 had the capacity to remove and prevent CRAB biofilms in a dose-dependent manner.

### Synergistic activity of colistin and phage using a microtiter plate assay

First, the antimicrobial activity of colistin against the bacterial host strain was evaluated. The minimum inhibitory concentration (MIC) and minimal bactericidal concentration (MBC) values of colistin against CRAB ABPW052, the host of phage vWU2001 were 0.25 and 0.5 μg/mL, respectively. The synergistic activity of colistin and phage was evaluated. The combinations of phage (at an MOI of 0.1 or 1) plus colistin at 1/16 MIC (0.0156 μg/mL) and 1/2 MIC (0.125 μg/mL) were considered MIC and MBC, respectively. The synergy activity was calculated by checkerboard analysis. The fractional inhibitory concentration (FIC) index value was 0.06, which indicates synergism^32^.

### Growth-kill curve of the combination of phage and colistin

**Figure 6:**
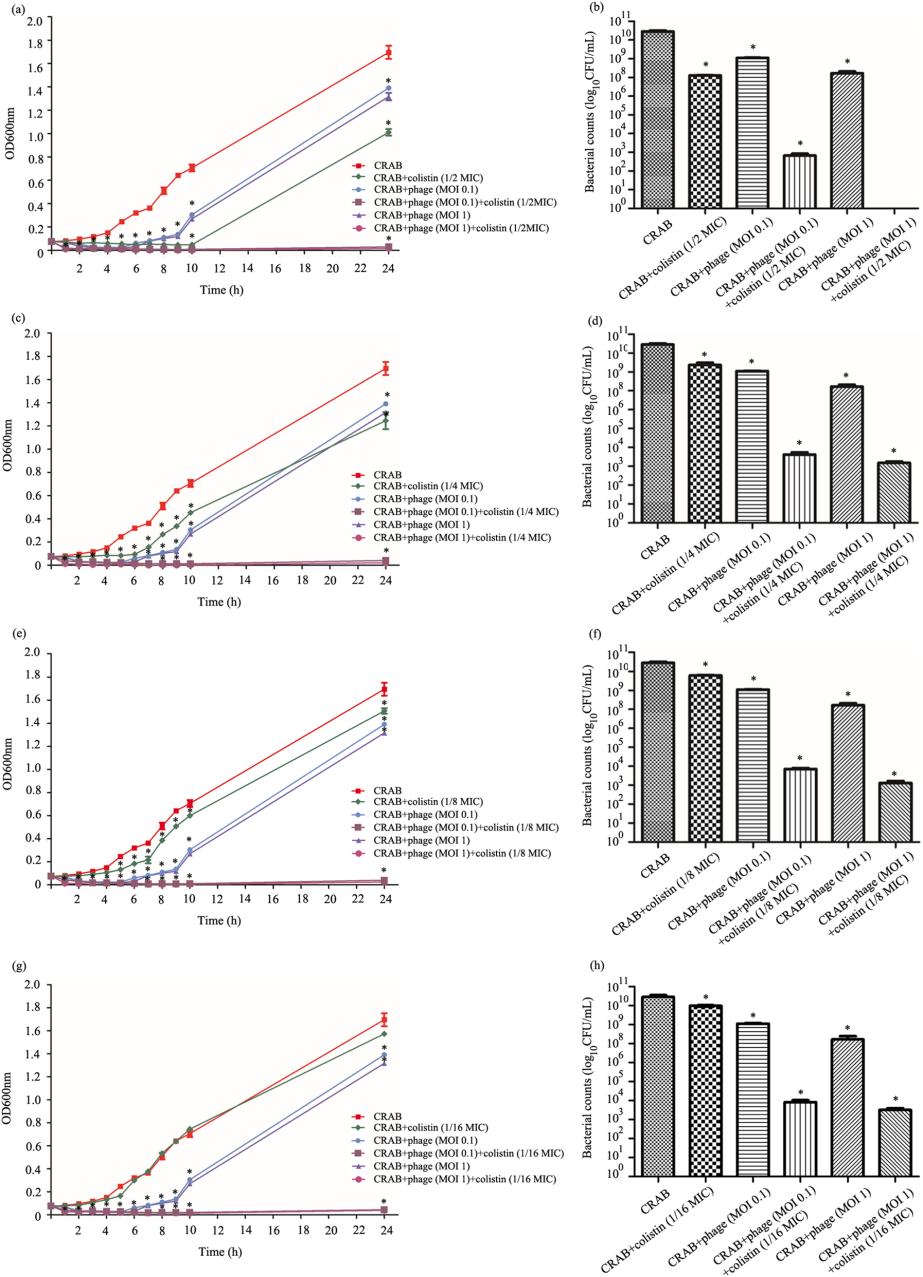
Synergistic antibacterial activity of phage vWU2001 and colistin against CRAB. Effect of the combination of phage and colistin at 1/2 MIC (**a**, **b**), 1/4 MIC (**c**, **d**), 1/8 MIC (**e**, **f**), and 1/16 MIC (**g**, **h**) on the CRAB growth curve and cell viability, respectively. Experiments were undertaken independently in triplicate with duplicate assay. The data show the mean ± SD (*, *P* value < 0.05).

The synergistic effect of the combination of phage and colistin was further evaluated by a growth kill assay. The antimicrobial effects of phage vWU2001-alone (MOI of 0.1 or 1), colistin-alone (1/2 MIC-1/16 MIC), the combination (phage vWU2001 at an MOI of 0.1 and colistin), and the combination (phage vWU2001 at an MOI of 1 and colistin) were determined every hour for 10 h to obtain a bacterial growth kill curve. At 24 h, the growth curve and bacterial cell viability were assessed. For a control, the growth of CRAB was observed. At 2 h post incubation, phage at MOIs of 0.1 and 1 significantly inhibited the growth of CRAB, but the results showed an increase in bacterial growth from 6 to 24 h (Fig. 6a,c,e,g). However, at 24 h post infection, CRAB growth and bacterial cell viability of treatments with only phage were significantly lower than those of the control (*P* ≤ 0.002). The phage at an MOI of 1 showed significantly higher bactericidal capability than the phage at an MOI of 0.1 (*P* ≤ 0.0083). For colistin treatment alone, colistin at 1/2 MIC to 1/8 MIC significantly inhibited the growth curve at 10 h. Nevertheless, the results showed the induction of CRAB growth and bacterial cell viability at 24 h. Both combinations (phage at an MOI of 0.1 and colistin, and phage at an MOI of 1 and colistin) significantly reduced CRAB growth at 1 h post incubation (*P* ≤ 0.0002). At 24 h, the combinations showed a significantly higher efficacy in reducing the CRAB growth curve and CRAB cell viability, as defined by a ≥ 5 log CFUs/mL decrease in viable bacterial counts when compared with phage or colistin treatment alone at all tested concentrations (*P* ≤ 0.0195) (Fig. 6b,d,f,h). The combination of phage at an MOI of 1 and colistin showed a greater reduction in CRAB growth than the combination of phage at an MOI of 0.1 and colistin (*P* ≤ 0.0220)*.*

### Structural details of bacterial cells

**Figure 7:**
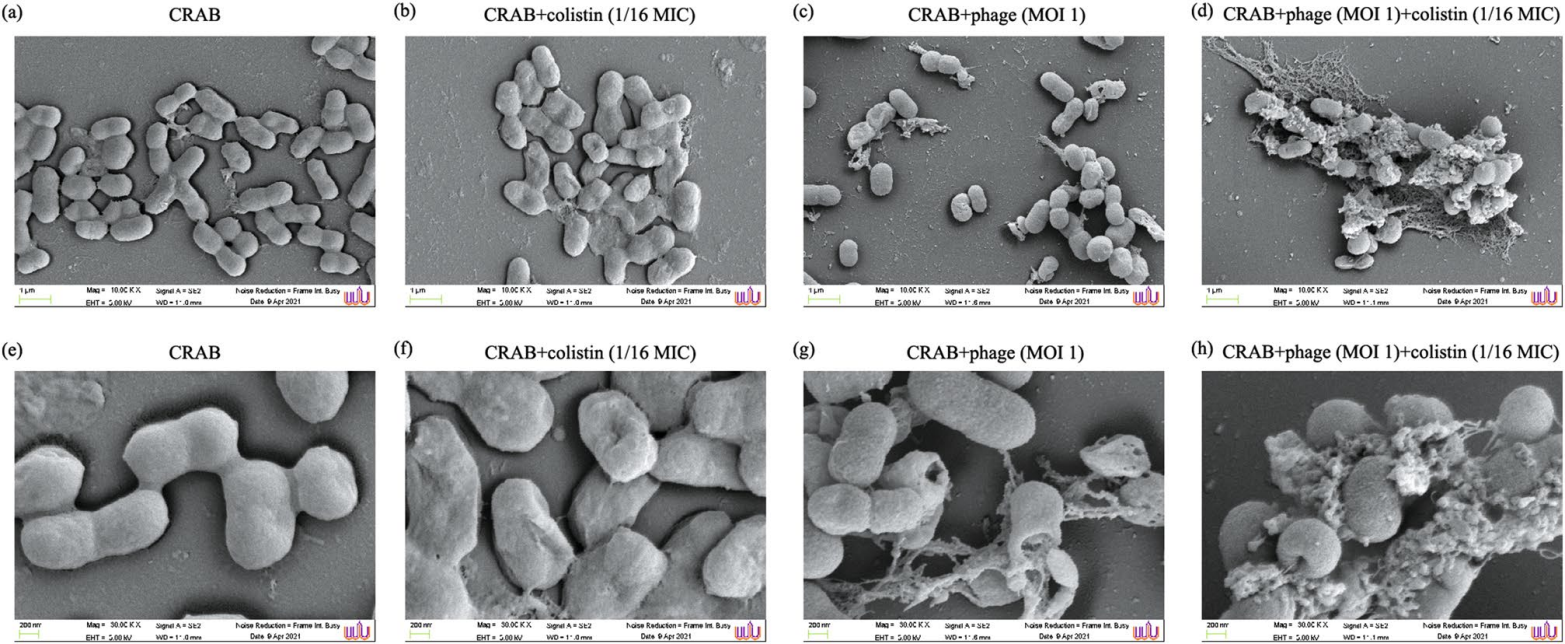
Structural details of CRAB cells under SEM. (**a**, **e**) CRAB; (**b**, **f**) CRAB treated with colistin at 1/16 MIC; (**c**, **g**) CRAB treated with phage at an MOI of 1; (**d**, **h**) CRAB treated with the combination of phage and colistin.

The bacterial cell morphology after treatment with only colistin, only phage vWU2001, and co-treatment of phage vWU2001 and colistin was investigated under a scanning electron microscope. CRAB had a coccobacillus form with a smooth surface (diameter 0.5–0.6 nm and length 0.9–1.3 μm) (Fig. 7a,e). For the 1/16 MIC colistin treatment only, a slight disruption of the CRAB cell membrane was seen in some cells (Fig. 7b,f). CRAB cell-treated with phage or a combination of phage and colistin induced wrinkled surfaces, protrusions, cellular shrinkage, membrane blebbing, cavitation and pore formation of the bacterial membrane, leading to disruption of the cell membrane and bacterial cell death (diameter 0.5–0.7 nm and length 0.6–0.7 μm). Cell contents were released to the exterior of the cells, and cellular debris accumulated around the cells (Fig. 7c,d,g,h). The bacterial cell numbers in the only phage vWU2001 treatment and co-treatment with phage vWU2001 and colistin were reduced when compared to the number of CRAB cells in the control; no treatment, and only colistin treatment samples (Fig. 7a,b,c,d). The number of bacterial cells was reduced more in the treatment with the combination of phage and colistin than in the only phage treatment (Fig. 7c,d).

### In vivo synergistic effect of phage and colistin

**Figure 8:**
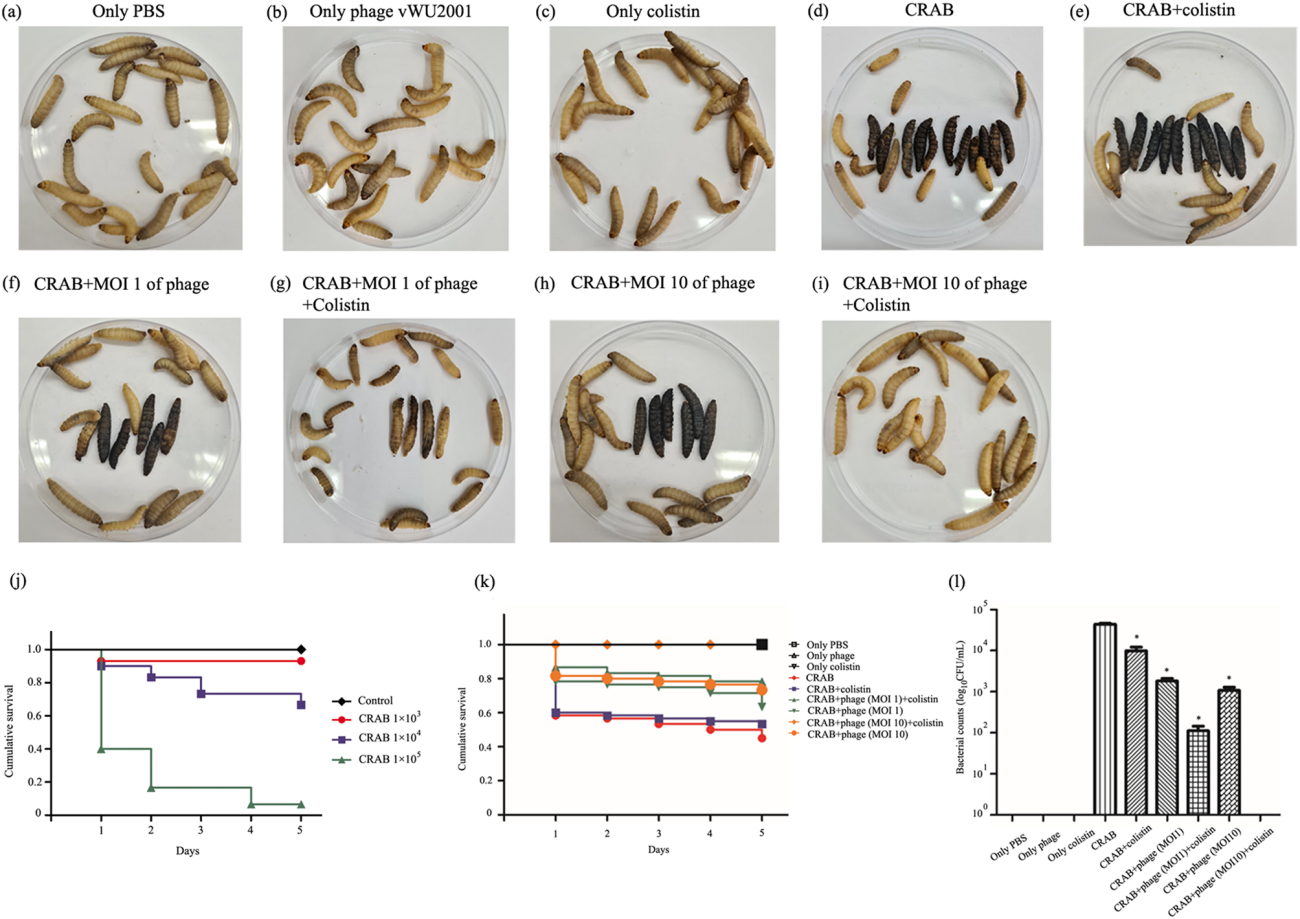
Synergistic effect of phage vWU2001 and colistin in CRAB infected *G. mellonella.* (**a**–**i**) Morphology of *G. mellonella*. If the larvae moved and did not show signs of melanization, they were considered alive. If the larvae had signs of melanization and did not move in response to touch, they were considered dead; (**j**) survival rate of *G. mellonella* larvae on establishment of CRAB infection; (**k**) survival rate of *G. mellonella* larvae on treatment; (**l**) bacteria count in *G. mellonella* larvae at day 5 post infection. Experiments were undertaken independently in triplicate (10 larvae per group) with duplicate assay. The data show the mean ± SD (*, *P* value < 0.05).

The synergistic effect of phage vWU2001 and colistin in *G. mellonella* larvae was evaluated. To determine an appropriate infective dose of bacteria, the larvae were injected with various concentrations of CRAB and mortality was observed for 5 days. The mortality rate of larvae increased when the concentration of bacteria increased (Fig. 8j). The LD_50_ dose at 5 days post infection, 4.3 × 10^4^ CFU/mL, was used in further studies. To determine any synergistic effects *G. mellonella* larvae were treated with a combination of phage and colistin followed by morphological observation. The survival of larvae was recorded daily and the bacterial number was counted at the end of the experiments. At 5 days post-infection, the control groups, which were larvae treated with only PBS, phage, and colistin did not induce *G. mellonella* larval melanization and death, indicating that PBS, phage at an MOI of 10, and colistin at 1/16 MIC were safe for larvae (Fig. 8a,b,c). Larval melanization and death were observed in CRAB infected *G. mellonella* larvae (Fig. 8d). The Kaplan–Meier survival curve showed that approximately 45% larval survival was recorded (Fig. 8k). Treatment of infected larvae with only colistin did not prevent melanization (Fig. 8e). Approximately 53% of larvae survived after treatment with only colistin, and the number of bacteria significantly decreased by nearly 2 log (Fig. 8k, l). Treatment with phage vWU2001 at MOIs of 1 and 10 reduced larval melanization and death (Fig. 8f,h). The survival rates of larvae were 63 and 73% in infected larvae treated with MOIs of 1 and 10, respectively (Fig. 8k). The higher MOI of phage vWU2001 showed greater efficacy in larval treatment. For the combination groups, the combination of colistin and phage at an MOI of 1 reduced larval melanization, while no melanization was observed with the combination of colistin and phage at an MOI of 10 (Fig. 8g,i). Larvae treated with the combination of colistin and phage at MOIs 1 and 10 showed 79% and 100% viability, respectively (Fig. 8k). The number of bacteria in larvae was significantly decreased by approximately 2.3 log with the combination of phage at an MOI of 1 and colistin (*P* < 0.0001). No viable bacteria were observed in larvae treated with the combination of colistin and phage at an MOI of 10 (Fig. 8l). The results indicate that the combination of phage and colistin showed significantly higher efficacy in reducing melanization and increasing the survival rate of larvae than the phage alone, or colistin treatment alone.

## Discussion

Antibiotic-resistant *A. baumannii* is one of the most important nosocomial pathogens worldwide, causing substantial morbidity and mortality. *A. baumannii* is frequently involved in hospital-acquired pneumonia (HAP) and ventilator-associated pneumonia (VAP). Both HAP and VAP are associated with the increasing prevalence of resistant nosocomial pathogens. The resistance rate of *A. baumannii* isolated from hospitals to antibiotics has risen rapidly, including carbapenem resistance^33–35^. Carbapenems constitute an important class of antibiotics for the treatment of nosocomial infections caused by gram-negative bacteria^36^. Carbapenems consist of many different agents such as doripenemb, ertapenem, imipenem, imipenem/cilastatin, meropenem, and piperacillin/tazobactam. In recent years, *A. baumannii* has become resistant to nearly all antibiotics, so new drugs, and alternative treatments, including phage therapy, are necessary to develop. This study characterized a novel lytic phage vWU2001 specific for CRABs, followed by evaluation of the antibacterial and synergistic activities of phage and colistin in vitro and in vivo.

Phage vWU2001 was isolated from the wastewater samples after treatment. Phage vWU2001 formed large clear plaques with a halo zone on a lawn of the host strain, indicating the presence of phage-encoded depolymerase degrading polysaccharide capsules. The phage host range results indicated that phage vWU2001 might have a broad host range activity, supporting the possibility of its use in phage therapy. However, an increase in the number of bacterial strains in determination of phage host range will be useful to confirm the broad range of phage vWU2001. Phage vWU2001 adsorbed efficiently to the host CRAB. Moreover, the phage displayed a short latent period and a large burst size, indicating that it could produce high progeny in a short time period of time. Suppression of CRAB growth in liquid culture also indicated a strong bacteriolytic effect of phage vWU2001.

Environmental factors for phage storage are the main factors, influencing phage activity. Phage vWU2001 tolerated a broad range of temperature and pH values. UV radiation is a major cause of phage inactivation in the environment^37,38^. The viability of phage vWU2001 was decreased after exposure to UV which is similar to other phages^39,40^. When comparing phage viability, phage vWU2001 was more resistant to UV irradiation than *Klebsiella* phage KP1801^40^ and *Dickeya* phage BF25/12^39^. Moreover, phage vWU2001 survived in long-term storage. Based on the outstanding biological characteristics of phages, phage vWU2001 could be a promising alternative to overcome CRAB infection.

The criteria recommended by ICTV were used to demarcate the new species of phage. The sequences identity was determined using BlastN and EMBOSS Stretcher analysis. The DNA sequence identity compared to other phages was less than 95%, indicating that phage vWU2001 is a novel species. Based on sequence homology the phage was closely related to *Acinetobacter* phage phiAB6, and the genome analysis showed that phage vWU2001 was a member of the realm *Duplodnaviria*, kingdom *Heunggongvirae*, phylum *Uroviricota*, class *Caudoviricetes*, order *Caudovirales*, family *Autographiviridae*, subfamily *Beijerinckvirinae*, and genus *Friunavirus*^41^. There were no virulence genes and lysogeny-related genes, such as integrase, recombinase, repressor genes, or excisionase, in the genome, indicating that phage vWU2001 should be considered a safe lytic phage. RNA polymerase and tail fiber genes were selected to investigate the genetic relationships between phages. RNA polymerase is a unique enzyme among phages^42^. The phylogenetic tree of RNA polymerase was constructed to confirm the relatedness of the phage. The RNA polymerase of phage vWU2001 shared some sequence similarity to the RNA polymerase of *Acinetobacter* phage AbKT21phiIII. The identity of RNA polymerase sequences demonstrated that phage vWU2001 was grouped into the genus *Friunavirus*, corresponding to the comparative whole genome analysis. Previous studies have reported that tail fiber proteins are involved in phage adsorption and penetration into bacterial cells^43^. Tail fibers bind specifically to the surface of the host bacterial strain^44^, and differences in tail fiber protein might affect host binding, so the phylogenetic tree of tail fiber might provide insights to understanding the host range. Tail fiber proteins of *Acinetobacter* phage IME200^45^ and phage phiAB6^46^ possessed a depolymerase activity, which could combat biofilms and infection^47^. In addition, the degradation of capsular polysaccharide by tail fiber protein allows the penetration of phage into the bacterial cell surface^48^. Thus, the use of depolymerase is an interesting approach for the treatment of bacterial infections^45^. Based on the plaque morphology of phage vWU2001, the halo zones of plaques which are often used as an indicator of depolymerase activity were observed^49^. Moreover, the ORF34 of phage vWU2001 encoded tail fiber gene product and the ORF34 was subjected to analyze the function by InterProScan^50^. Amino-acid positions 225–432 of the tail fiber protein matched with domains of pectate lyase (PF12708 and IPR024535). The pectate lyase domain has been previously proposed as having a function in phage depolymerase activity and this domain binds specifically to the *Acinetobacter* bacterial capsule^51^. Another report showed that the Dpo7 protein-containing pectin lyase domain was also expressed as EPS depolymerases^52^. The results indicated that phage vWU2001 had depolymerase activity that can degrade bacterial capsular polysaccharides and exopolysaccharides. Multiple alignments of tail fiber proteins, proteins related to host recognition, showed high similarity to other phages^53^, but differences between sequences were observed. Therefore, phage vWU2001 might possess a different host range than other phages. All phages were grouped in the *Friunavirus* genus, corresponding to whole genome and RNA polymerase analysis.

Biofilms, one of the virulence factors contributing to *A. baumannii* infection, are bacterial communities. Biofilms enhance antibiotic resistance, leading to an increase in a wide variety of chronic infections, prolonged hospital stays, death, and economic loss^54^. Biofilms are surrounded by the EPS matrix which is a barrier to antibiotic diffusion, so bacteria in biofilms are generally more resistant to current treatments^55^. Some studies on the ability of *Acinetobacter* phages to prevent biofilms have been reported. Phage ISTD-specific CRAB reduced the number of biofilm viable cells at 6 h post incubation^17^. Phage AB7-IBB1 at an MOI of 10^5^ eradicated more than 75% of biofilms of *A. baumannii* in both polystyrene and human embryonic kidney (HEK) 293 cell lines^56^. According to the results of genomic analysis, the tail fiber protein, which has been reported to possess depolymerase activity, was encoded by phage vWU2001. The efficacy of phage treatment on biofilms was investigated. Phage vWU2001 showed great potential in biofilm removal and prevention of biofilm formation, suggesting that it is possible to use phage vWU2001 as an alternative biocontrol agent.

Some bacteria have developed resistance to phage infection through different pathways such as spontaneous mutations, adsorption resistance, penetration blocking systems, receptor blocking, and adaptive immunity associated with CRISPR/Cas systems^57,58^. Bacterial resistance against phage has been reported in several previous studies. Regrowth of CRAB and MDR *A. baumannii* was detected after treatment with phage ISTD and phage vB_AbaM-IME-AB2 at 3 and 4 hpi, respectively^17,59^. A similar phenomenon was observed in our lytic activity study. The regrowth of CRAB against phage vWU2001 was detected. The absorbance of bacteria resistant to phage was still lower than that of untreated CRAB. The results indicated that CRAB was slightly resistant to phage vWU2001 attack. The rapid emergence of phage-resistant bacteria has limited the use of phage^60^.

Colistin is a membrane-destabilizing agent that induces membrane permeability in gram-negative bacteria^61^. Although colistin is as an important post-antibiotic agent with high efficiency, it has been used as a last line of treatment due to its side effects, such as nephrotoxicity and neurotoxicity^62^. Higher colistin doses might be associated with the induction of convulsions^63^. In addition, a high antibiotic dosage also impacts the increase in bacterial mutations and resistant bacteria, leading to antibiotic treatment failure^64,65^. Reducing antibiotic doses can slow the evolution and emergence of antibiotic-resistant bacteria^66,67^. Only a few studies on the synergistic relationship between *Acinetobacter* phages and antibiotics have been undertaken^21^. To completely eradicate bacterial infections and suppress bacterial resistance against phages, the combination of phage and colistin, the last resort antibiotic to treat *A. baumannii*, was evaluated. Phage vWU2001 combined with colistin at 1/2 MIC to 1/16 MIC had a synergistic effect on CRAB in vitro*.* The combination of phage and colistin effectively inhibited CRAB proliferation and suppressed the emergence of bacterial resistance. To further confirm the synergistic activity in vivo, the combined treatment was assessed in *G. mellonella* larvae. A few studies on phage-antibiotic combinations in *G. mellonella* larvae have been reported. *Acinetobacter* phage vB_AbaP_AGC01 showed synergism with ciprofloxacin and meropenem^68^. The synergistic effect of polymyxin B and *Acinetobacter* phages WCHABP1 or WCHABP12 was assessed, but no consistent synergism was observed^69^. The combination of phage vWU2001 and colistin in infected larvae treatment was more effective in removing CRAB than phage or antibiotic alone. The development of phage-resistant CRAB could be prevented by the co-addition of colistin. Interestingly, our results showed that the combined treatment of phage vWU2001 and colistin could improve the survival rate of larvae and that the treatment completely cleared CRAB in larvae. The results confirmed that the synergistic phage-colistin combination could be an advantage over using phages or antibiotics alone.

The mechanism of the phage-antibiotic combination is still unclear. The action of colistin on the outer membrane of gram-negative bacteria might enhance both phage adsorption and phage DNA injection^21^. A previous study has indicated that the synergism of phage and antibiotic effected bacterial morphological changes, leading to rapid phage maturation and acceleration of cell lysis^70^. Based on our SEM micrographs, cells developed a cell clustering or clustered arrangement in the presence of colistin. Kamal and colleagues suggested that the clustered arrangement of bacterial cells induced the ability of phage to travel on the adjoined cell surface, increasing phage infection efficiency^71^. Efflux pumps play an important role in inducing the resistance of *A. baumannii* to carbapenem and colistin^72^*.* It has been shown that phages could change an efflux pump mechanism, the cause of bacterial resistance to phages in *P. aeruginosa*, leading to restoration of antibiotic sensitivity^73^. There have been reports of the ability of phage to slow or reverse the emergence of antibiotic-resistance^73^. Phages might provide an adjuvant effect and the emergence of resistant cells could be suppressed by synergism^74^. Moreover, some phages showed increase plaque size in the presence of antibiotics, and antibiotics could increase phage production^71^. It has been reported that antibiotics could stimulate phage development in *E.coli* and *S. aureus*^75,76^. Higher numbers of phage in the presence of antibiotics could enhance the antibacterial activity, promoting a synergistic antibacterial effect^77^. The combined approach could also lead to the induction of phage production in larvae^71^. Several reports showed that the sequential treatments might be related to the efficacy of synergistic. When the phage was given first, the bacteria adapted themselves to escape from the phage. This escape step might increase the susceptibility of bacteria to antibiotics, leading to synergistic activation^73^. However, other conditions, such as dose concentrations, time of treatment, and route for administration, are related to efficacy^78^.

In conclusion, a novel *Acinetobacter* phage, vWU2001, has the potential to be used as an alternative antibacterial agent to control CRAB infections in vitro and in vivo. The phage vWU2001-antibiotic combination might be an important approach to combat CRAB*.* Further evaluation of the identification of bacterial genotypes specific to phage vWU2001, the antibiofilm activity of the combinations, and a mammalian model will be assessed.

## Materials and methods

### Bacterial strains and growth conditions

Forty-two clinical isolates of CRAB*, E. coli, K. pneumoniae*, *MRSA,* and *P. aeruginosa* were originally isolated as routine laboratory specimens from Songklanagarind Hospital, Prince of Songkla University, Songkhla Province, Thailand. All bacterial isolates were kindly provided by the Natural Product Research Center of Excellence, Prince of Songkla University*. A*. *baumannii* ATCC 17978 was included. For maintenance, bacteria were cultured on tryptic soy agar (TSA; Becton, Dickinson and Company, Franklin Lakes, NJ) at 37 °C for 24 h. For liquid preparations, bacterial colonies on TSA were transferred to 3 mL of sterile tryptic soy broth (TSB; Becton, Dickinson and Company, Franklin Lakes, NJ) or Mueller Hinton Broth (MHB; Becton, Dickinson and Company, Franklin Lakes, NJ). The bacterial suspension was then incubated at 37 °C with continuous shaking at 150 rpm. For long-term preservation, bacterial isolates were kept in sterile 40% (*v*/v) glycerol solutions at − 80 °C.

### Phage enrichment and isolation

Wastewater samples after water treatment by a surface aeration system were collected from a local hospital, Thasala hospital, Nakhon Si Thammarat, Thailand. CRAB strain ABPW052 was employed as a host for phage isolation and propagation. After the removal of bacterial cells and sample debris by centrifugation (6000 × g, 10 min, 4 °C) and filtration (a sterile 0.22 μm filter (Merck Millipore, Burlington, MA)), 10 mL of the filtrate was mixed with 10 mL of TSB and 200 μL of exponential-phase bacterial culture for phage enrichment. The mixture was incubated in a shaking incubator at 150 rpm overnight at 37 °C. After incubation, the culture was centrifuged at 6000 × g for 20 min at 4 °C and then filtered through a sterile 0.22 μm filter. The supernatant was collected for phage detection and enumeration by the double-layer agar method.

### Conventional double-layer agar method

Phage was serially diluted in SM buffer (0.1 M NaCl, 8 mM MgSO_4_.7H_2_O, 50 mM Tris–HCl pH 7.5) and 200 μL of the phage diluted solution was mixed with 200 µL of log-phase bacterial culture. The mixture was mixed with top agar (0.75% agar in TSB) and then quickly poured onto a TSA plate. After incubation overnight at 37 °C, plaques were observed.

### Phage purification and amplification

Phage was purified four times by the double-layer agar technique. A single plaque was picked from the lawn of host bacteria and then soaked in 500 μL SM buffer. The plaque was left at 4 °C overnight in SM buffer and then centrifuged at 6000 × g for 10 min at 4 °C. After filtration by a sterile 0.22 μm filter, the filtrate was serially diluted in SM buffer. The supernatant was collected to perform the double-layer agar method for observing the plaque morphology. To obtain the purified phage, these steps were repeated four times. For phage amplification, the purified phage was propagated in the bacterial host. The phage supernatant from the fourth time of phage purification was serially diluted in SM buffer. Two hundred microliters of the diluted phage was mixed with 200 μL of exponential-phase bacterial culture followed by the double-layer agar method to obtain the semi-confluent plates. Five milliliters of SM buffer was added to the plates for eluting phage. After incubation at 4 °C overnight, the supernatant was collected and then centrifuged at 6,000 × g for 20 min at 4 °C followed by filtration using a sterile 0.22 μm filter. The filtrate was kept as the phage stock at 4 °C until used. The phage titer was determined using standard plaque assay as plaque forming unit (PFU)/mL.

### Phage morphological characterization by transmission electron microscopy (TEM)

Phage morphology was determined as described previously^40^. Twenty microliters of the phage suspension was spotted onto a carbon-coated copper grid followed by negative staining with 2% (*v*/v) uranyl acetate (pH 6.7). The grids were air-dried and the phage was observed under a JEM 2010, JEOL Germany electron microscope at an operating voltage of 160 kV.

### Phage host range analysis

Host range was evaluated against 42 clinically isolated CRAB*, A*. *baumannii* ATCC 17978, *E. coli, K. pneumoniae*, *MRSA,* and *P. aeruginosa* strains. The standard spot test was conducted to determine the lytic activity of the phage as previously described^40^. Two hundred microliters of exponential-phase clinically isolated CRAB was mixed with top agar and then poured onto a TSA plate. Ten microliters of the phage solution (10^4^ PFU/mL) were dropped onto top agar and allowed to air-dry. After incubation overnight at 37 °C, the lytic zone was observed. Experiments were undertaken in duplicate.

### Efficiency of plating of phage (EOP)

EOP was performed to quantify the lytic activity of the phage as previously described^79^. Twenty-three bacterial isolates were selected based on their sensitivity against phage vWU2001, including the bacterial host strain. The phage was serially diluted in SM buffer, and 100 µL of the serial phage dilution was then mixed with 200 µl of each log-phase bacterial isolate (MOI = 0.01). To attach the phage to the host, the mixtures were incubated for 15 min, and the plaque-forming ability of the phage was assessed by the double agar overlay method. The EOP was calculated as the ratio of the number of virus particles infecting the test bacterium to the number of virus particles infecting the host bacterium. Experiments were undertaken independently in duplicate with duplicate plaque assay.

### Phage adsorption rate assay

CRAB bacteria were mixed with the phage at an MOI of 1 followed by incubation at 37 °C without shaking. The mixtures were collected every 5 min for 30 min and then centrifuged at 12,000 × g for 5 min at 4 °C. Supernatants were collected and filtered through a 0.22 μm pore size filter. The phage titer was determined by the double agar layer assay. Experiments were undertaken independently in duplicate with duplicate plaque assay.

### The rate of attachment of phage to cells

The adsorption rate constant* k* was measured followed the previous report^25^. CRAB bacteria were diluted and then infected with phage vWU2001. The supernatant was collected every minute until 10 min and the supernatant was immediately added into the pre-chilled TSB supplemented with chloroform. The phage titer was determined by the double agar layer assay. The control was medium supplemented with phage. Experiments were undertaken independently in duplicate with duplicate plaque assay. The adsorption rate constant *k*, in ml/min was calculated.

### One-step growth curve

The phage latent period and burst size were inferred by a one-step growth curve as previously described^40,80^. Briefly, exponential-phase CRAB was centrifuged at 6000 × g for 20 min at 4 °C. After discarding the supernatant, the pellet was resuspended in TSB and then infected with phage at an MOI of 1 by incubation for 15 min. The unabsorbed phage was removed by centrifugation at 6000 × g for 20 min at 4 °C. The pellet was resuspended in TSB and then incubated at 37 °C with shaking at 150 rpm. The samples were collected at 10 min intervals for 120 min followed by phage titration by the soft-agar overlay method. The latent period was the time between phage adsorption and the release of phage progeny from infected bacterial host cells. The burst size, the number of phage progeny released by an infected cell, was calculated as the ratio of the released phage progeny to the initial count of infected bacterial host cells during the latent period. Experiments were undertaken independently in duplicate with duplicate assay.

### Isolation of phage-resistant mutant strain

The phage-resistant mutant strain was isolated as previously described^81^. Exponential-phase clinically isolated CRAB was mixed with the phage supernatant at MOI of 10. After incubation at 37 °C for 24 h, the bacterial suspension was diluted in TSB and then spread on TSA plates followed by incubation at 37 °C overnight. Single colonies were isolated and streaked on TSA plates for purifying a single and pure colony. A spot test was used to confirm the phage-resistant mutant strain and the colony confirmed to resist phage vWU2001 infection was kept as the phage-resistant mutant strain. Gram staining and biochemical tests were performed.

### In vitro phage activity against CRAB and phage-resistant mutant strain

A killing curve was generated to determine the lytic activity of the phage against CRAB and phage-resistant mutant strain^40,82^. Briefly, CRAB and the phage-resistant mutant strain were infected with the phage at MOIs of 0.01, 0.1, 1 and 10. The mixtures were incubated in a shaking incubator at 37 °C at 150 rpm and the OD600 values were measured every hour for 24 h using a UV-spectrophotometer. CRAB or phage-resistant CRAB mutant strain without the phage was used as a control sample. Experiments were undertaken independently in duplicate with duplicate assay.

### Effect of temperatures, pH values, and UV radiation on the phage stability

The stability of phage vWU2001 was evaluated at different temperatures, pH values, and UV radiation levels according to previously employed methods^40^. The phage stock was diluted in SM buffer to 10^8^ PFU/mL and then incubated at different temperatures (− 80 °C, − 20 °C, 4 °C, 25 °C, 37 °C, 40 °C, 50 °C, 60 °C, 70 °C and 80 °C). Two hours post incubation, the phage suspensions were cooled slowly in an ice-water bath, and phage stability was assessed by the double-layer agar method. To determine the effect of pH on phage stability, phage were exposed to different pH values (1–14) for 2 h, and phage at different pH values were neutralized to pH 7. The phage stability was determined by the double-layer agar method. Phage incubated at pH 7 and 37 °C were used as a control. The phage stability was assessed under UV radiation. Phage in open Petri dishes were incubated on ice, placed 30 cm away from the UV light source, and then exposed to a UV-C light at the intensity 1.722 ± 0.112 w/cm^2^. The phage suspension was collected every 10 min for 1 h followed by the double-layer agar. Experiments were undertaken independently in duplicate with duplicate plaque assay.

### Stability of phage in a glycerol stock

The stability of phage vWU2001 in glycerol stock was evaluated. The phage supernatant was supplemented with glycerol to a final concentration of 25% (v/v) and 50% (v/v)^83^. The samples were incubated at − 20 °C and − 80 °C and the stability of phage was determined by the double-layer agar method at day 7 post incubation. Experiments were undertaken independently in duplicate with duplicate plaque assay.

### Long term stability

The phage suspension in SM buffer (10^8^ PFU/mL) was stored at 4 °C up to 6 months to determine long-term phage stability. The viability of the phage was determined every month by preparing ten-fold serial dilutions in SM buffer and the titer of the phage was evaluated by the double agar overlay method. Experiments were undertaken independently in duplicate with duplicate plaque assay.

### Whole genome characterization and analysis

The phage whole genome de novo sequencing was determined commercially on the Illumina sequencing platform (Macrogen Inc., Seoul, South Korea). Briefly, DNA of phage vWU2001 was extracted and then subjected to the library preparation using TruSeq Nano DNA library preparation kit (Illumina, California, USA). Briefly, DNA was mechanically fragmented to generate DNA fragments with 3' or 5' overhangs by Covaris shearing with the Covaris M220 focused-ultrasonicator (Covaris, Woburn, MA). The DNA fragments were subjected to end repair, size selection, and A-tailed. The ends of the DNA fragments were ligated to adapters. To amplify the amount of DNA in the libraries, the adapter-ligated DNA fragments were enriched by PCR. Liberies were quantified using a fluorometric quantification method and library quality was checked using an Agilent Technologies 2100 Bioanalyzer. DNA libraries were normalized and pooled. Subsequently, the library was sequenced. The quality of raw sequence data from high throughput sequencing pipelines was checked by FastQC (version 0.11.5). The filtered reads were assembled into a contig by SPAdes de novo (version 3.15.0) with k-mer size 123 bp. The sequence coverage of the phage genome was analyzed by the Bowtie2 mapper provided by Geneious R10^84^. The locations of protein coding sequences, tRNA genes, tmRNA genes, and rRNA genes were predicted by Prokka (v1.12), and the functions were then annotated by BLAST and RAST server^85^. BlastX was performed against standard databases with the E-value cutoff of 0, the query coverage cutoff of 70%, and the identity cutoff of 60%. The amino acid sequences were further searched to predict proteins on the Phyre2 web server^86^. InterProScan was used to identify protein domains^50^. The genomic termini and the terminal repeats were predicted based on in silico identification by PhageTerm^87^. The genome map was constructed on the CGview server. To analyze the new species of phage vWU2001, ViPtree and EMBOSS Stretcher analysis were used to assess the genomic synteny and sequence identity, respectively.

The ViPTree, viral proteomic tree server was used to construct the viral proteomic trees^88^. The phage nucleotide genome sequences were uploaded using the default setting and the genome was searched against prokaryote host categories. To analyze the relationship of whole genome sequences, the phage genome was searched for closely related phage by BlastN search. The E-value cutoff of 0, the query coverage cutoff of 70%, and the identity cutoff of 60% were used. The outgroup was *Vibrio* phage vB_VhaP_VH-5 (MN497414.1). The Genome-BLAST Distance Phylogeny (GBDP) method was used to analyze all pairwise comparisons of the nucleotide sequences under settings recommended for prokaryotic viruses^89–91^. The intergenomic distances were used to infer a balanced minimum evolution via FASTME including SPR post-processing^92^. Branch support was inferred from 100 pseudo-bootstrap replicates each. The tree was constructed and visualized using FigTree^93^. The OPTSIL program^94^, the recommended clustering thresholds^91^ and an F value (fraction of links required for cluster fusion) of 0.5^95^ were used to analyze the taxon level such as species, genus and family.

### Phylogenetic analysis of specific genes

RNA polymerase and tail fiber genes were selected to investigate the genetic relationships by the maximum-likelihood phylogenetic tree and JTT matrix-based model based on the amino acid sequence alignment. Both genes were compared with other phages sequences in the NCBI database by BlastX. The multiple sequence alignments were performed with MUSCLE and the maximum likelihood phylogenetic trees were constructed in MEGA-X using 1000 bootstrap replicates.

### Biofilm assay

The efficacy of phage vWU2001 on the prevention of biofilm formation and biofilm removal was investigated according to a method described previously^40^. The biofilm biomass and cell viability were evaluated in parallel. To study the effect of phage on biofilm formation, bacterial suspensions were introduced into a flat-bottomed 96-well microtiter plate followed by the addition of 100 μL of the indicated numbers of phage (10^1^–10^8^ PFU/well). The plates were incubated without agitation at 37 °C for 48 h. At the indicated time points, the contents of the wells were aspirated, and then washed twice with PBS. The microplates were air-dried and then stained with 200 μL of 0.1% crystal violet for 30 min. The excess stain was washed with PBS and the biofilm was solubilized with 200 μL of absolute ethanol. The biofilm biomass was assessed by measuring the OD595 using a standard microplate absorbance reader. The biofilm viable cell numbers were determined by colony counting. The medium was removed, and the biofilm was washed twice with PBS. One hundred microliters of PBS was added to the wells, and the biofilm cells were suspended by vigorous pipetting. The suspended biofilm was transferred into a new flat-bottomed 96-well microtiter plate and was diluted tenfold by PBS. Ten microliters of this solution was dropped onto TSA plates and then incubated at 37 °C overnight. For preformed biofilm eradication, bacterial suspensions were added into a flat-bottomed 96-well microtiter plate and allowed to form biofilms. Following incubation at 37 °C for 48 h, the bacterial suspension was removed and the biofilm was then washed twice with PBS. One hundred microliters of the indicated numbers of phages (10^1^–10^8^ PFU/well) was inoculated and incubated at 37 °C for 24 h. At the indicated time points, biofilm biomass and biofilm viable cell numbers were quantified as described above. Experiments were undertaken independently in triplicate with duplicate assay.

### Determination of the antibacterial activity of colistin and the combination of phage and colistin against CRAB

The minimal inhibitory concentration (MIC) and minimal bactericidal concentration (MBC) of colistin against CRAB were determined, followed by an evaluation of colistin and phage synergism. A modified broth microdilution method was performed according to Clinical and Laboratory Standard Institute (CLSI) guidelines^96,97^. Briefly, CRAB was cultured at 37 °C until the log phase was reached. The bacterial culture was adjusted to match the turbidity of the 0.5 McFarland standard (1 × 10^8^ CFU/mL) and the bacterial suspension was then diluted to 1 × 10^6^ CFU/mL. Colistin (Siam Pharmaceutical Co. Ltd., Bangkok, Thailand) was added to the plate and then underwent twofold serial dilutions in MHB. Subsequently, 100 µL of bacterial suspension was added to the plate, containing 50 µl serially diluted colistin and 50 µl MHB. The plates were incubated at 37 °C for 18 h. To determine the MIC value, ten microliters of resazurin (Sigma-Aldrich Chemicals, St. Louis, Missouri, USA) was added to the wells. The plates were then incubated in the dark for 2 h and changes in color were observed. The lowest concentration prior to color change was considered the MIC value, and concentrations greater than or equal to the MIC value were used to assess the MBC value. Ten microliters of the broth at each concentration was dropped onto Mueller Hinton Agar (MHA; Becton, Dickinson and Company, Franklin Lakes, NJ) plates. Colony growth was observed after incubation at 37 °C overnight. The lowest concentration at which no growth was considered the MBC value. Experiments were undertaken independently in triplicate with duplicate assay.

To determine the synergistic antibacterial activity of phage in combination with colistin against CRAB, colistin was serially diluted in the wells of microtiter plates followed by the addition of 50 µL of phage at an MOI of 0.1 or 1 diluted in MHB. One hundred microliters of diluted CRAB suspension was inoculated into the wells and incubated at 37 °C for 18 h. Antibacterial activities of phage alone (MOI of 0.1 or 1), and colistin alone were assessed in parallel. MIC and MBC were determined as described above. Experiments were undertaken independently in triplicate with duplicate assay. The FIC index value was calculated by the following equation: ∑FIC = FIC of antibiotic + FIC of phage = MIC of colistin in combination/MIC of colistin alone + MIC of phage in combination/MIC of phage alone. The results were interpreted according to FIC indexes as follows: synergistic (∑FIC: ≤ 0.5), additative (∑FIC: > 0.5 and ≤ 1), indifferent (∑FIC: > 1 and ≤ 4), and antagonistic (∑FIC: > 4)^32^.

### Killing kinetics of the antibacterial activity of the phage and colistin combination

The effect of the combination of phage vWU2001 and colistin on the bacterial growth curve was assessed in 96-well plates and compared to the phage and colistin treatment alone effects. CRAB was cultured at 37 °C overnight and then adjusted to match the turbidity of the 0.5 McFarland turbidity standard. Fifty microliters of colistin at 1/2 to 1/16 MIC value was added to the microtiter plate wells followed by inoculation of 50 µL of phage at a MOI of 0.1 or 1. Subsequently, 100 µL of diluted bacterial suspension (1 × 10^6^ CFU/mL) was added to the wells and then incubated at 37 °C. Bacterial growth was measured at OD600 nm every hour for 10 h followed by incubation overnight. At 24 h post incubation, the OD600 nm was measured and the number of viable bacterial cells was counted by bacterial colony counting. A tenfold dilution series of bacterial suspension was prepared in TSB and ten microliters of the diluted suspension was dropped onto TSA plates which were incubated at 37 °C overnight. Experiments were undertaken independently in triplicate with duplicate assay.

### Scanning electron microscopy (SEM)

The efficacy of phage vWU2001, the colistin, and the combination of phage and colistin on CRAB cells were visualized under SEM. Briefly, CRAB was incubated with only phage at an MOI of 1, colistin (1/16 MIC), or the combination of phage (MOI = 1) and colistin (1/16 MIC), individually. After incubation at 37 °C for 2 h, the samples were centrifuged at 12,000 × g for 5 min, and the supernatant was discarded. The pellets were washed twice with PBS and then fixed with 2.5% glutaraldehyde in 0.1 M PBS. After washing with 0.1 M sodium phosphate buffer, the cells were incubated in 1% OsO_4_ in DI water. The cells were washed with DI water followed by dehydration in a series of ethanol solutions (20%, 40%, 60%, 80%, and 100%). The cells were dried in a critical point dyer and incubated in a desiccator for 24 h. The cells were coated with gold and observed under a field emission scanning electron microscope (Merlin compact, Zeiss, EDX (Oxford, Aztec), EBSD (Oxford, Nordlys Max)).

### Synergistic phage-antibiotic combinations for the control of CRAB in *G. mellonella*

The animal experiments were approved by the Animal Ethics Committee, Walailak University, Thailand (The animal ethics approval certificate (number: WU-AICUC-64–002)). Subsequently, the animal testing was performed. *G. mellonella* larvae were used as an in vivo model for evaluating the efficacy of the combination of phage vWU2001 and colistin therapy against CRAB infections. The surface of healthy larvae weighing 300 mg were swiped with 70% ethanol. Larvae were injected in the last left pro-leg with 20 µl of CRAB diluted in 10 mM PBS pH 6.5 (1 × 10^3^, 1 × 10^4^, and 1 × 10^5^ CFU/mL). Only PBS was injected for the negative controls. Larvae were placed into Petri dishes and then incubated at 37 °C in darkness under a humidified atmosphere, with honey for 5 days. Survival and melanization scoring were carried out daily. If the larvae moved and did not show signs of melanization, they were considered alive. If the larvae had signs of melanization and did not move in response to touch, they were considered dead. A set of 10 larvae per group was used. A half-maximum lethal dose (LD50) was calculated for the synergistic study. Experiments were undertaken independently in triplicate with duplicate assay.

To evaluate the effect of phage and colistin combination on CRAB infection, larvae were injected with CRAB followed by inoculation with the combination of phage and colistin. Briefly, larvae were infected with 20 μL of CRAB at the LD50 dose into the hemolymph. At 2 h postinfection, the larvae were then inoculated with 20 μL of phage in PBS buffer at an MOI of 1 or 10, in combination with colistin at 1/16 MIC on the side opposite to the CRAB injection site on the larvae. PBS only, phage (MOI 10), and colistin injections were used as negative controls. Survival and melanization of larvae were recorded daily for 5 days. At the end of the experiments, the CRAB viability in larvae was evaluated by a colony counting assay. Larvae were ground in ice-cold PBS and the supernatant was serially diluted in PBS. The diluted bacterial supernatant was dropped onto TSA plates and the bacterial number was determined by counting CFUs after overnight incubation. Experiments were undertaken independently in triplicate with duplicate assay.

### Statistical analyses

All data was analyzed using the GraphPad Prism program, version 5 (GrapPad Software, https://www.graphpad.com/scientific-software/prism/). Statistical analysis of significance was undertaken by unpaired T-test using the GraphPad Prism, *P* < 0.05 for significance. The freeware ED50plus (v1.0) software was used to calculate LD50 value (http://sciencegateway.org/protocols/cellbio/drug/data/ed50v10.xls).

## Supplementary Information

Supplementary Information 1.

Supplementary Information 2.
