# Supplementary material for: Enhanced antibacterial effect of a novel Friunavirus phage vWU2001 in combination with colistin against carbapenem-resistant Acinetobacter baumannii
Source: Sci Rep. 2022 Feb 16;12:2633. doi: 10.1038/s41598-022-06582-0 (PMC8850435; doi:10.1038/s41598-022-06582-0)
Supplement: Supplementary file 1 — Supplementary Information 1. [file 41598_2022_6582_MOESM1_ESM.pdf]

Enhanced antibacterial effect of a novel *Friunavirus* phage vWU2001 in combination with colistin against carbapenem-resistant *Acinetobacter baumannii*

Phitchayapak Wintachai[1*], Narumon Phaonakrop[2], Sittiruk Roytrakul[2], Ampapan Naknaen[3], Rattanaruji Pomwised[3], Supayang Piyawan Voravuthikunchai[3,4], Komwit Surachat[5], Duncan R Smith[6]

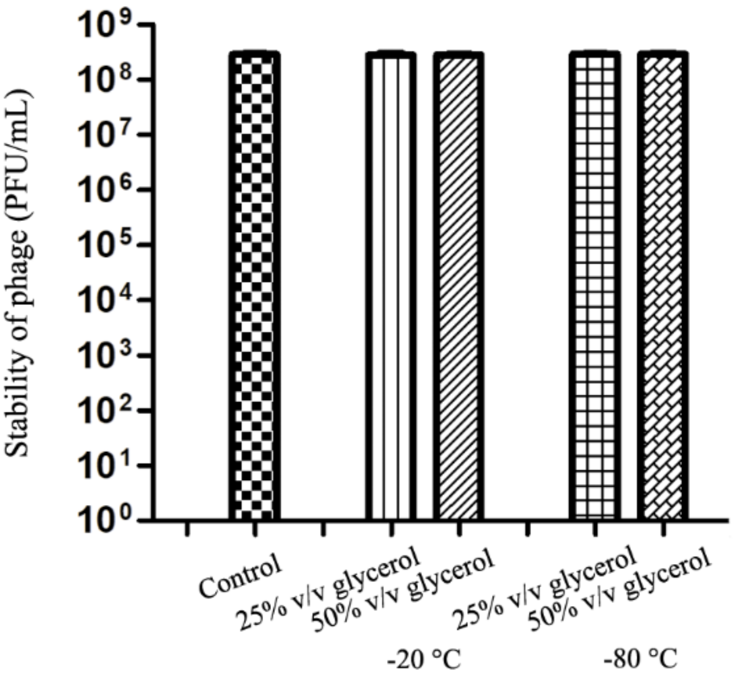

Supplementary Figure S1. Stability of phage vWU2001 in different concentrations of glycerol, a cryoprotective agent at -20 and -80 °C. Experiments were undertaken independently in duplicate with duplicate assay. The data show the mean±SD (*, *P* value<0.05).

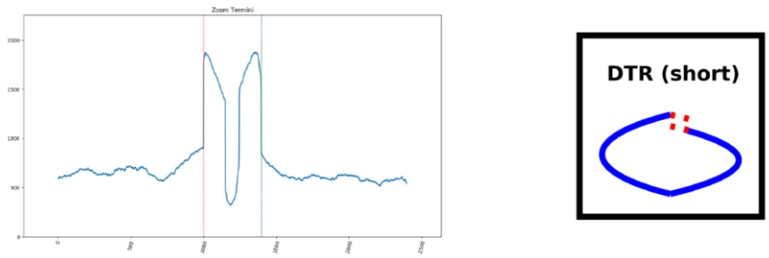

PhageTerm Method

Ends	Left (red)	Right (green)	Permuted	Orientation	Class	Type
Redundant	36729	37125	No	NA	DTR (short)	T7

**Direct Terminal Repeats: 397 bp*

Supplementary Figure S2. PhageTerm analysis of phage vWU2001

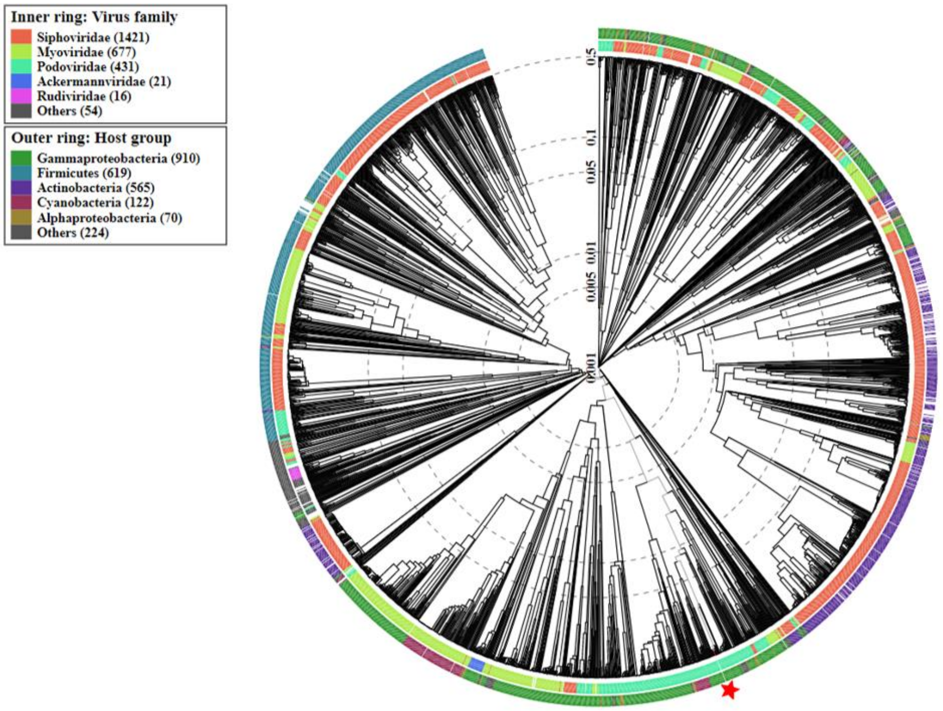

Supplementary Figure S3. Phage phylogenetic tree. Protein-level phylogenetic tree, generated by VIPtree using a protein distance metric based on normalized tBLASTx scores plotted on a log scale. The tree included 2688 phages. Phage vWU2001 was highlighted with a red star.

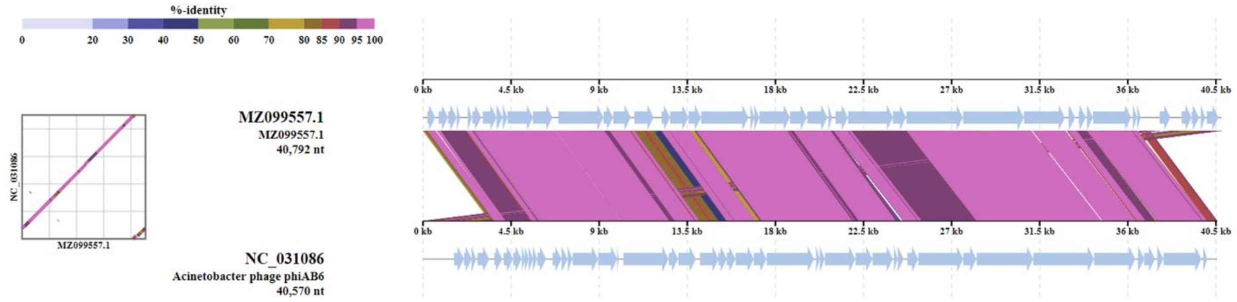

Supplementary Figure S4. Genome alignment of phage vWU2001 and *Acinetobacter* phage phiAB6, generated by VIPtree. All tBLASTx alignments are represented by colored lines between two genomes. Color scale represents The tBLASTx percent identity was presented by color scales.
